# Role of PKR in the Inhibition of Proliferation and Translation by Polycystin-1

**DOI:** 10.1155/2019/5320747

**Published:** 2019-06-23

**Authors:** Yan Tang, Guang Shi, JungWoo Yang, Wang Zheng, Jingfeng Tang, Xing-Zhen Chen, Jianzheng Yang, Zuocheng Wang

**Affiliations:** ^1^Department of Oncology, The Second Hospital, Jilin University, Changchun 130041, China; ^2^Membrane Protein Disease Research Group, Department of Physiology, Faculty of Medicine and Dentistry, University of Alberta, Edmonton, Canada T6G2H7; ^3^National “111” Center for Cellular Regulation and Molecular Pharmaceutics, Hubei University of Technology, Wuhan 430086, China

## Abstract

Autosomal dominant polycystic kidney disease (ADPKD) is mainly caused by mutations in the PKD1 (~85%) or PKD2 (~15%) gene which, respectively, encode polycystin-1 (PC1) and polycystin-2 (PC2). How PC1 regulates cell proliferation and apoptosis has been studied for decades but the underlying mechanisms remain controversial. Protein kinase RNA-activated (PKR) is activated by interferons or double-stranded RNAs, inhibits protein translation, and induces cell apoptosis. In a previous study, we found that PC1 reduces apoptosis through suppressing the PKR/eIF2*α* signaling. Whether and how PKR is involved in PC1-inhibited proliferation and protein synthesis remains unknown. Here we found that knockdown of PKR abolishes PC1-inhibited proliferation and translation. Because suppressed PKR-eIF2*α* signaling/activity by PC1 would stimulate, rather than inhibit, the proliferation and translation, we examined the effect of dominant negative PKR mutant K296R that has no kinase activity and found that it enhances the inhibition of proliferation and translation by PC1. Thus, our study showed that inhibition of cell proliferation and protein synthesis by PC1 is mediated by the total expression but not the kinase activity of PKR, possibly through physical association.

## 1. Introduction

Autosomal dominant polycystic kidney disease (ADPKD) is one of the most common inherited renal diseases and characterized by the development of fluid-filled cysts [1, 2]. Up to 95% of the ADPKD cases are caused by mutations in the PKD1 or PKD2 gene which encodes polycystin-1 (PC1) and polycystin-2 (PC2), respectively. Mutations in PKD1 account for ~85% of ADPKD [3, 4]. PC1 is a 462-kDa membrane protein with 4302 amino acids (aa) in length, eleven transmembrane (TM) domains, a large extracellular N-terminus and a short C-terminus containing domains involved in G-protein activation and interaction with partner proteins [5–7]. PC1 seems to function as a cell surface receptor that mediates mechanosensation of fluid flow of primary cilia in renal epithelia and intracellular signalling [8–10].

ADPKD is a disorder characterized by several cellular abnormalities, including cell overproliferation, apoptosis, and dedifferentiation [11], which indicates a high cell turnover rate. It was reported that cyclic adenosine monophosphate (cAMP) and mitogen-activated protein kinase (MAPK) signaling [12], P53, c-Jun N-terminal kinase(JNK) induction [13] and activation of cellular Src kinase (c-Src) [14], signal transducers and activators of transcription (STAT) [15], Hippo [16], and *β*-catenin/Wnt pathway [17] are connected with overproliferation or differentiation of renal epithelial cells in ADPKD. In cyst-lining epithelial cells of ADPKD patients and mouse model experiments, the mammalian target of rapamycin (mTOR) pathway was shown to be activated, which may result from loss of PC1 binding with tuberin, suggesting that PC1 inhibits cell proliferation by downregulating mTOR activity through interaction with tuberin [18]. Actually, PC1 reduces the cell growth by negatively regulating mTOR and downstream effectors ribosomal protein S6 kinase beta-1(S6K1) and eukaryotic translation initiation factor 4E-binding protein 1 (4EBP1) in a tuberin-dependent manner [18, 19]. mTOR-related translational control pathways have then been subjects of study with respect to PKD pathogenesis [20–25]. Despite tremendous progress made in the development of ADPKD pathogenesis over the past years, the underlying mechanisms are still elusive.

Protein kinase RNA-activated (PKR) is a serine/threonine protein kinase that is activated by interferons, double-stranded RNAs (dsRNAs), cytokine, growth factor, and stress signals [26]. The 551-aa kinase is made up of a C-terminus for catalysis as well as a N-terminus which contains double-stranded RNA of approximately 70aa residues each; when combined with dsRNAs, the conformation of PKR changed, and the binding site dimerized to form PKR dimer [10]. During virus infection, PKR is activated and blocks viral protein synthesis through phosphorylation of eukaryotic translation initiation factor 2 alpha (eIF2*α*), thus leading to antiviral defense [27]. PKR can be autophosphorylated with the formation of dimer and activated when binding to dsRNA, and then phosphorylates substrates, including eIF2*α*, protein phosphatase 2A (PP2A), and inhibitor of nuclear factor kappa-B(I*κ*B) kinase (IKK). PKR inhibits translation and promotes apoptosis through the substrates and downstream effectors [28, 29]. In addition to PKR, there are three other eIF2*α* kinases, including protein kinase-like endoplasmic reticulum (ER) kinase (PERK), general control nonderepressible 2 (GCN2), and heme-regulated inhibitor (HRI) [30]. Phosphorylated eIF2*α* (P-eIF2*α*) blocks translation initiation but activates some selected proteins critical to cell survival, including transcription factor 4(ATF4), growth arrest, and DNA damage gene (GADD34) and C/EBP-homologous protein (CHOP) [31, 32].

Up to now, there has been no literature report on whether or how PKR mediates the inhibition of proliferation and translation caused by PC1. Therefore, the aim of this study was to investigate the involvement of PKR in PC1-regulated proliferation and translation.

## 2. Materials and Methods

### 2.1. Reagents and Antibodies

Puromycin was a product of Sigma-Aldrich Canada. Phosphorylated PKR (P-PKR, Thr446), P-PKR (pT446), PKR (B-10), and anti-FLAG antibodies were purchased from Santa Cruz (Santa Cruz, CA) or Epitomics (Burlingame, CA). eIF2*α*, P-eIF2*α*, and mTOR antibodies were products of Cell Signaling Technology (New England Biolabs, Pickering, ON). Anti-GFP (B-2) was from Santa Cruz and anti-GFP (EU4) from Eusera (Edmonton, AB). Mouse monoclonal anti-*β*-actin (C4, Santa Cruz) antibody was employed as loading controls. Secondary antibodies were from Santa Cruz or GE Healthcare (Baied'Urfe, QC).

### 2.2. DNA Constructs, Cell Culture, and Transfection

Plasmid pcDNA3-GFP-PC1-5TMC(PC1-5TMC, aa 3895-4302) comprising last 5 TMs plus C-terminus was constructed using Stratagene Quik Change® II XL Site-Directed Mutagenesis Kit (Agilent Technologies Canada Inc., Mississauga, ON) as described previously [33]. Plasmid eIF2*α* was from Santa Cruz (Santa Cruz, CA). All cDNA sequences of the constructed plasmids were verified by sequencing. Human embryonic kidney (HEK293T) or HeLa cells were grown in Dulbecco's modified Eagle's medium with 10% fetal bovine serum, penicillin-streptomycin, and L-glutamine in an atmosphere of 5% CO_2_ and 37°C. HEK293T cells with stable transfection of wild type (WT) mouse PC1 was from one coauthor Dr. J. Yang and cultured under the above conditions with 2*μ*g/ml of puromycin [34]. Transient transfection was performed on HEK293T or HeLa cells grown to ~70% confluency employing lipofectamine 2000 reagent (Invitrogen).

### 2.3. Gene Knockdown

Small Interfering RNAs (siRNA) of PKR (Santa Cruz, Cat#sc-36263) was utilized to interfere with HEK293T and HeLa cells according product description. HEK293T or HeLa cells at 50–60% confluency were transfected in normal culture medium without antibiotics, supplemented with Opti-MEM medium (Invitrogen, Burlington, ON) and lipofectamine 2000. 10 pmol of siRNA was added to the transfection reagent for 40 hours (hr). The efficiency of the siRNA knockdown was assessed by immunoblotting.

### 2.4. Cell Proliferation Assay

HEK293T or HeLa cells were transiently transfected with corresponding plasmids such as GFP, GFP-PC1-5TMC, PKR, and PKR siRNA in 100 mm dishes. At 24 hr after transfection, cells were seeded into either new 100 mm dishes for further transfection such as eIF2*α*, PKR, PKR-K296R, and PKR knockdown or a 96-well plate for alarmaBlue (Invitrogen Canada Inc.). After incubation for another 16-30 hr, absorbance was measured using a microplate reader (Fluoroskan Ascent FL, Thermo Labsystems). The rest of the cells in the 100 mm dishes were collected for immunoblotting at the same time point. HEK293T cells stably expressing WT PC1 were seeded in 100 mm dishes overnight and then transfected with PKR-K296R or PKR siRNA using 4*μ*l lipofectamine 2000 reagent in medium lacking serum. 6 hr after transfection, the plates were replenished with medium containing 10% serum and incubated at 37°C for an additional 24 hr before measurements. The cell proliferation rate (%) was calculated as OD_test_/OD_control_×100%.

### 2.5. ^35^S Pulse Labelling

HEK293T or HeLa cells were transfected with plasmids using Lipofectamine 2000 reagent. At 40 hr after transfection, equal number of cells was starved for 1 hr in the prelabeling medium (L-methionine and L-cysteine free DMEM with 10% FBS and penicillin/streptomycin, Invitrogen), followed by pulse labeling with 50 *μ*Ci of [^35^S] methionine/cysteine (EXPRE ^35^S Protein Labeling Mix, PerkinElmer, Woodbridge, ON) for 10 minutes, as described previously [35, 36]. Cell extracts were used for sodium dodecyl sulfate–polyacrylamide gel electrophoresis (SDS-PAGE) and autoradiography.

### 2.6. Coimmunoprecipitation (Co-IP)

Experiments were carried out according to previously established methods [37]. Briefly, HeLa cells (2×10^7^ cells) with plasmid pEGFP-PC1-5TMC or pEGFP transfection were collected for extraction of protein and immunoprecipitation at 40 hr after transfection. 20mg of the total protein was for immunoblotting and 200mg for co-IP.

### 2.7. Statistical Analysis

All data generated were presented as mean±standard error (SEM). N represents the number of repeat experiments. Data analyses were measured using Sigmaplot 12.0 software (Systat Software Inc., San Jose, CA). A P-value ⩽0.05 was statistically significant.

## 3. Results

### 3.1. Inhibition of Proliferation and Translation by PKR

**Figure 1:**
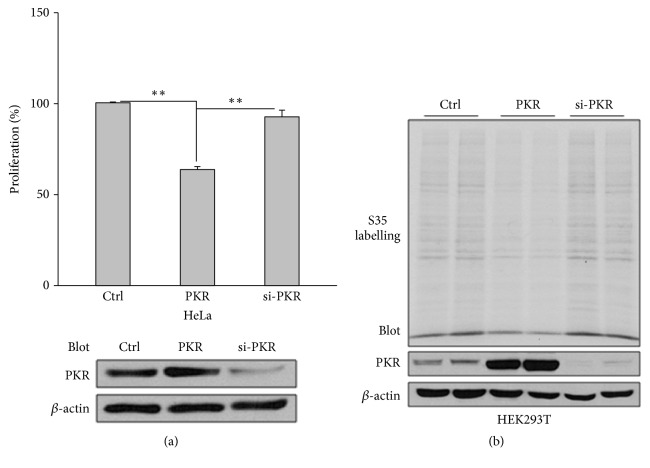
*Effects of PKR on the proliferation and translation*. (a) Effects of PKR on the proliferation of HeLa cells. After being transfected with plasmids PKR, PKR siRNA, or GFP, HeLa cells were plated in multiple wells of a 96-well plate and grown for 24 hr for cell proliferation assays. Cells from the sample preparations were collected for immunoblotting. Proliferation rate of the control sample was normalized to 100%. PKR, WT PKR; si-PKR, PKR siRNA; Ctrl, GFP. Upper panel, averaged data (N=4, *∗∗*p<0.01). Lower panel, effectiveness of transfection and siRNA of PKR assessed by immunoblotting. (b) Effect of PKR on protein synthesis in HEK293Tcells. HEK293T cells transiently transfected with GFP, PKR, or PKR siRNA were starved for 1 hr followed by pulse labelling for ^35^S pulse labelling assays followed by SDS-PAGE and immunoblotting assays with the antibody against total PKR. *β*-actin served as loading control.

We found that PC1 reduces apoptosis by inhibiting the PKR kinase activity and the phosphorylation of eIF2*α* [33]. Here we tested whether PKR is involved in PC1-inhibited proliferation. In order to clarify the effect of PKR on cell proliferation and translation, we used alarmaBlue to label HeLa cells for cell proliferation assays and performed ^35^S labelling assays in HEK293T to evaluate protein translation. We found that PKR suppresses proliferation and translation whereas PKR knockdown by siRNA does not show stimulation effect (Figures 1(a) and 1(b)), which is in line with previous reports [28, 29] and suggests that the endogenous PKR activity is not a rate-limiting factor for the proliferation and protein synthesis.

### 3.2. Independency of PC1-Mediated Inhibition of Proliferation from eIF2*α*

**Figure 2:**
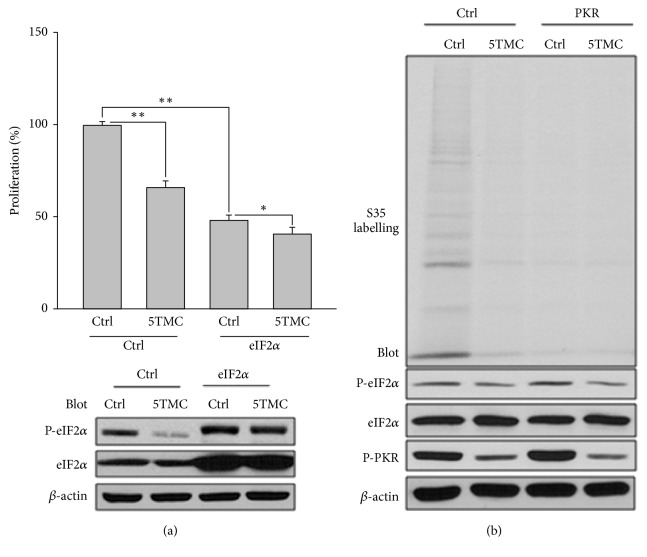
*Effects of PKR/eIF2α overexpression on PC1-5TMC-inhibited proliferation and translation in HeLa cells*. (a) Effects of eIF2*α* overexpression on PC1-5TMC-inhibited proliferation. Transfected with GFP or PC1-5TMC, HeLa cells were cotransfected with eIF2*α*. They were then plated in multiple wells of a 96-well plate and grown for 24 hr for cell proliferation assay; cells from the sample preparations were collected for immunoblotting. 5TMC, GFP-tagged PC1-5TMC; eIF2*α*, WT eIF2*α*; Ctrl, GFP. Upper panel, averaged data (N=4,*∗*p< 0.05, *∗∗*p< 0.01). Lower panel, effectiveness of transfection of eIF2*α* assessed by immunoblotting. (b) Effects of PKR overexpression on PC1-5TMC-inhibited protein synthesis. After transfected with GFP or PC1-5TMC, HeLa cells were cotransfected with WT PKR. They were then used for ^35^S pulse labeling assays followed by SDS-PAGE and immunoblotting assays with the antibodies against P-eIF2*α*, total eIF2*α*, and P-PKR. *β*-actin served as loading control.

We next examined whether PC1 and PKR-eIF2*α* inhibit cell proliferation or protein translation through the same pathway. Overexpression of PC1 truncate mutation encoding 5 TMs and C-terminus (PC1-5TMC, aa 3895-4302) inhibited cell proliferation of HeLa cells (Figure 2(a)). HeLa cells overexpressing eIF2*α* exhibited much reduced proliferation rates, as expected, and were still inhibitable by PC1-5TMC (Figure 2(a)), indicating that the eIF2*α* activity and PC1 inhibit proliferation through two different pathways. In fact, if inhibition by PC1 were through eIF2*α*, then because PC1-5TMC reduces the eIF2*α* activity [33], a known proliferation and translation inhibitor, we would see a stimulating effect of PC1-5TMC on proliferation, against our observation (Figure 2(a)). PC1-5TMC also inhibited protein synthesis assessed by ^35^S labelling, but because overexpressed PKR almost completely stopped ^35^S labelling, the effect of coexpressed PC1-5TMC on protein synthesis cannot be evaluated (Figure 2(b)).

### 3.3. Dependence of PC1-Inhibited Proliferation and Translation on Total PKR

**Figure 3:**
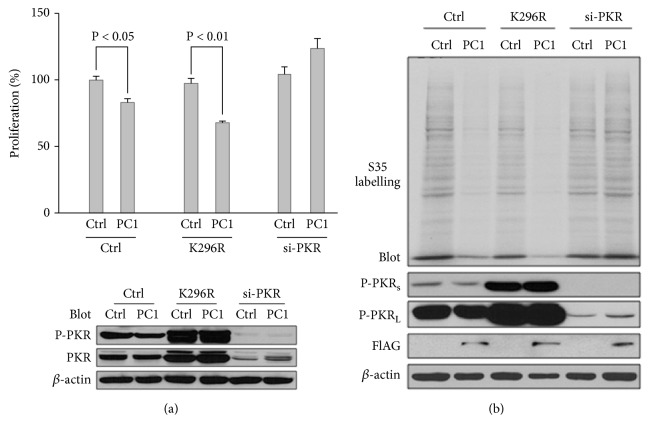
*Effects of PKR siRNA and PKR-K296R on PC1-inhibited proliferation and translation*. (a) Effects of PKR siRNA and PKR-K296R on PC1-inhibited proliferation. HEK293T and those stably transfected full-length PC1 were transfected with GFP vector, PKR-siRNA or PKR-K296R, and then plated in multiple wells of a 96-well plate and grown for 24 hr for cell proliferation assays. Cells from the sample preparations were collected for immunoblotting. PC1, GFP-tagged full-length PC1; Ctrl, GFP; K296R, PKR-K296R. Upper panel, averaged data (N=4). Lower panel, effectiveness of PKR-K296R transfection and PKR siRNA assessed by immunoblotting. (b) Effects of PKR-K296R and PKR siRNA on PC1-inhibited translation. HEK293T and those cells stably transfected full-length PC1 were transfected with GFP vector, PKR-siRNA, or PKR-K296R and then for ^35^S pulse labelling assays, followed by SDS-PAGE and immunoblotting assay with the antibody against P-PKR. *β*-actin served as loading control. P-PKRs, band with short exposure of 0.5 min; P-PKR_L_, band with common exposure of 2 min.

We further examined the role of PKR in PC1-inhibited proliferation and protein synthesis. When PKR was knocked down by siRNA, PC1 no longer inhibited proliferation of HEK cells, indicating the requirement of PKR for mediating the effect of PC1 (Figure 3(a)). Interestingly, expression of PKR-K296R that can retain the autophosphorylation of PKR but has lost kinase function [38] did not have significant effect on proliferation but allowed strong inhibition of proliferation by PC1 (Figure 3(a)). ^35^S labelling assays also showed that PKR knockdown abolishes while expression of mutant K296R rescues the inhibition of protein synthesis in HEK cells by PC1 (Figure 3(b)). Taken together, out data showed that PC1-inhibited proliferation and translation are mediated by a pathway that depends on the total PKR but not its kinase activity.

Based on the above results that phosphorylated PKR/eIF2*α* exerts an opposite effect on PC1-inhibited proliferation/translation and is not involved in PC1-inhibited proliferation/translation, we deduced that PC1 inhibits cell proliferation/protein translation through the total expression of but not the kinase activity of PKR.

### 3.4. Interaction of PC1 with PKR and mTOR

**Figure 4:**
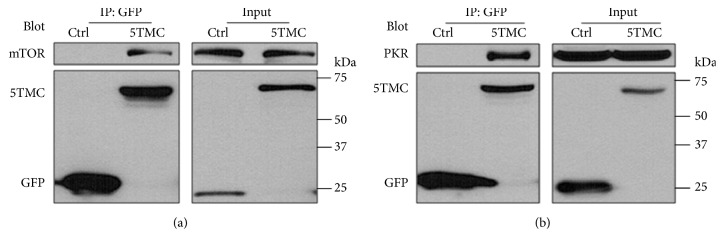
*Physical interaction of PC1 truncate mutation, PKR, and mTOR in HeLa cells*. (a) Interaction between mTOR and PC1-5TMC. 200mg of protein extracted was immunoprecipitated with antibody against GFP (EU4), and 20mg of protein extracted was detected by immunoblotting assays with antibody against GFP (B-2). The blot was reprobed with anti-mTOR. 5TMC, GFP-tagged PC1-5TMC; Ctrl, GFP. (b) Interaction between PKR and PC1-5TMC. Experiments performed are similar to those in panel (a). The blot was reprobed with anti-PKR.

Dependence of PC1-inhibited proliferation and translation on the total PKR suggested that PC1 may inhibit proliferation and translation by physical protein-protein interaction. It is well known that PC1 reduces cell size by negatively regulating mTOR and downstream molecules [18, 19]. Furthermore, it was found that mTOR and PKR may regulate the expression of PP2A subunit B56*α* independently of their kinase activity [39], while results from our current experiments showed that siRNA of B56*α* also abolishes PC1-inhibited proliferation and translation (data not shown). Therefore, we carried out co-IP experiments to document the physical interaction of PC1 with mTOR and PKR, and found that PC1-5TMC is in the same complex with mTOR and PKR in HeLa cells (Figures 4(a) and 4(b)), which is in line with previous reports [33, 40]. The results suggested that the PC1-PKR-mTOR association may mediate the effect of PC1 on proliferation and translation.

## 4. Discussion

Studies have shown that both PC1 and PC2 inhibit cell proliferation [19, 35]. Our previous study found that PC2 downregulates cell proliferation by promoting phosphorylation of eIF2*α* through increasing the efficiency of PERK [35]. We thus wondered whether eIF2*α* also takes part in PC1-inhibited proliferation. PKR is a well-known eIF2*α* kinase and is involved in various cellular functions. In particular, PKR as a kinase phosphorylates downstream substrates through which it regulates proliferation, translation, and apoptosis [28, 29]. In our previous study, we found that PC1 and PC1 truncate mutation inhibit P-eIF2*α* through reducing kinase activity of PKR [33]. Therefore, it is likely that the PC1-inhibited cell proliferation should be through a signaling that is different from PKR-eIF2*α* pathway, because suppressed PKR-eIF2*α* activity would promote cell proliferation and protein translation. However, surprisingly, PKR knockdown abolished and expression of nonfunctional PKR resumed the regulation of proliferation and translation by PC1. Together with the results of physical interaction of PC1-5TMC with PKR and mTOR, our data indicated that total PKR but not its kinase activity mediates the inhibition of proliferation and translation by PC1, possibly through physical association.

PC1 reduces cell growth by downregulating mTOR and downstream effectors in a tuberin-dependent manner [18, 19]. Because S6 and 4EBP1 are the well-known substrates of mTOR, while PP2A was shown to regulate translation initiation through dephosphorylating 4EBP1 and ribosomal protein S6 kinase beta-1(p70^s6k^) [40], we consider that PP2A may act as a major mTOR phosphatase to regulate downstream effectors. Furthermore, function of PP2A relies on B56*α*, an important member of regulatory B subunit families [41]. Ruvolo et al. considered that although mTOR regulates translational and transcriptional pathways by kinase activity, it does not directly regulate B56*α* by such pathways because a proteasome inhibitor can restore expression of the B subunit while PKR can protect B56*α* by suppressing proteasome-mediated proteolysis [39]. Based on our present experiment result of B56*α* knockdown also abolishing PC1-inhibited proliferation and translation (data not shown), we speculate the possibility that knockdown of PKR may inactivate PP2A and abolish PC1-dependent inhibition of proliferation/translation through promoting B56*α* proteolysis.

Through its protein-binding domain, PKR can act as an adaptor protein but not its regulatory dsRNA-binding domain [42, 43]. It was recently reported that protein-binding function of PKR promotes the proliferation of pancreatic *β* cells through TNF receptor-associated factor 2 (TRAF2)/receptor-interacting protein 1 (RIP1)/nuclear factor kappa-light-chain-enhancer of activated B cells (NF-*κ*B)/c-Myc pathway [44], while cancer cell survival requires mTOR-dependent phosphorylation of 4EBP1 in Myc-dependent tumor, and PP2A-B56*α* holoenzyme can negatively regulate c-Myc protein accumulation [45, 46].

Further experiments are needed to elucidate the mechanisms of involvement of total PKR, mTOR, PP2A-B56*α*, Myc, or other proteins in regulation of proliferation/translation by PC1.

## 5. Conclusions

In summary, our data showed that PKR knockdown by siRNA abolishes the inhibitory effect of PC1 on proliferation and translation, suggesting the dependence of the inhibition on PKR. Dominant negative PKR mutant K296R that retains the auto-phosphorylation ability but has no kinase activity increases the inhibition supported that PC1-inhibited proliferation and translation are mediated by the total but not the kinase activity of PKR. PC1-5TMC physically interacted with PKR and mTOR, which further indicated that the PKR-dependent inhibition of proliferation/translation by PC1 may be through physical association. Our study thus unveiled a novel mechanism of PC1-inhibited proliferation and translation that may be important for understanding ADPKD pathogenesis.

## Data Availability

The data used to support the findings of this study are available from the corresponding author upon request.
